# A 15-year review of dengue hospitalizations in Singapore: Reducing admissions without adverse consequences, 2003 to 2017

**DOI:** 10.1371/journal.pntd.0007389

**Published:** 2019-05-15

**Authors:** Li Wei Ang, Tun-Linn Thein, Yixiang Ng, Irving Charles Boudville, Po Ying Chia, Vernon Jian Ming Lee, Yee-Sin Leo

**Affiliations:** 1 National Centre for Infectious Diseases, Singapore; 2 Public Health Group, Ministry of Health, Singapore; 3 Department of Infectious Diseases, Tan Tock Seng Hospital, Singapore; 4 Lee Kong Chian School of Medicine, Nanyang Technological University, Singapore; 5 Saw Swee Hock School of Public Health, National University of Singapore, Singapore; 6 Yong Loo Lin School of Medicine, National University of Singapore, Singapore; Institute for Disease Modeling, UNITED STATES

## Abstract

**Objectives:**

Since the 1990s, Singapore has experienced periodic dengue epidemics of increasing frequency and magnitude. In the aftermath of the 2004–2005 dengue epidemic, hospitals refined their admission criteria for dengue cases to right-site dengue case management and reduce the burden of healthcare utilization and negative outcomes. In this study, we describe the national trends of hospital admissions for dengue and disease severity in terms of length of stay (LOS), admission to the intensive care unit (ICU) and death in hospital, and case fatality rate (CFR) in Singapore.

**Methods:**

We conducted a retrospective study of notified cases and laboratory confirmed dengue patients admitted to all public and private hospitals between 2003 and 2017. Case notifications for dengue and hospitalization records were extracted from national databases.

**Results:**

The proportion of dengue cases hospitalized was lower in recent years; 28.9% in the 2013–2014 epidemic, compared to 93.2% in the 2004–2005 epidemic, and 58.1% in the 2007 epidemic. Median LOS remained stable over the years; overall LOS was 3 to 4 days and ICU stay was 2 to 3 days. Less than 2% of hospitalized patients were admitted to the ICU. Overall CFR was low and remained below 0.5%. The proportions of dengue cases hospitalized and patients admitted to the ICU were highest in the elderly aged 65 years and older.

**Conclusions:**

While the proportion of dengue cases hospitalized saw a drastic decline due to more selective admission criteria, there was no concomitant increase in adverse outcomes, suggesting that admission criteria were appropriate to focus on severe dengue cases. Further studies are needed to optimize dengue management in older adults who are more likely to be hospitalized with greater disease severity, given the higher proportions of hospitalizations and severe disease among older adults.

## Author summary

Following the review of hospital admission criteria for dengue cases in the aftermath of the 2004–2005 dengue epidemic in Singapore, the proportion of dengue cases hospitalized plummeted from 72.6% in 2006 to the nadir of 25.6% in 2014. There was no concomitant increase in adverse outcomes as a result of the more selective admission criteria. Median length of stay remained stable over the years; overall stay was 3 to 4 days and intensive care unit (ICU) stay was 2 to 3 days. Among hospitalized dengue patients, less than 2% were admitted to the ICU. Overall case fatality rate was low and remained below 0.5%. Elderly patients aged 65 years and older constituted the highest proportions of dengue cases hospitalized and patients admitted to the ICU.

## Introduction

Dengue was ranked by the World Health Organisation (WHO) as the “most important mosquito-borne viral disease in the world” in 2012, in view of its increasing spread into previously unaffected areas and its high disease burden [1]. The global incidence of dengue has increased 30-fold over the past 50 years, and an estimated 2.5 billion people are at risk of infection, with dengue virus (DENV) endemic in over 100 countries spanning the Americas, Caribbean Basin, Asia and Africa [2]. The WHO Southeast Asia Region and Western Pacific Region are the most seriously affected, and together they contribute about three-quarters of the global dengue disease burden [3]. Singapore, a globally-connected island city-state, is one of several countries with high disease burden of dengue [4].

It is widely recognized that dengue epidemics impose a substantial burden on public health and health services, and incur considerable economic, societal and personal costs. In a local study, the annual average disease burden of dengue in Singapore was estimated to be 9–14 disability-adjusted life-years per 100,000 population during the ten-year period from 2000 to 2009, while the average economic impact of dengue illness ranged from $0.85 billion to $1.15 billion in 2010 US dollars [5]. To tackle the public health impact of dengue in Singapore, a comprehensive nationwide *Aedes* prevention and control programme incorporating environmental management and source reduction, health education and law enforcement was launched in 1969 and successfully implemented since 1973, as evidenced by a sharp reduction in the *Aedes* house index (percentage of residential premises found to be breeding *Aedes* mosquitoes) and low disease incidence [6,7]. Despite these efforts, dengue epidemics of increasing magnitude and an elevated level of endemicity have occurred since the late 1980s in a five- to six-year cycle [8–10].

The WHO, in laying out the global strategy for dengue prevention and control, has cited the particular challenges arising from unexpected surges in dengue cases, as well as the strain on health services arising from over-admission because of the limitations of triage in reliably predicting which severe cases will require hospital care [1]. The hospitalization rates of persons suspected of dengue viral infection are high, as doctors tend to err on the side of caution and admit them for monitoring. This leads to higher bed occupancy rates, although majority of dengue cases are unlikely to require or benefit from medical care in hospital for their mild form of illness.

In the aftermath of the 2004–2005 dengue epidemic in Singapore, hospitals reviewed and refined their admission criteria for dengue cases [11–13]. The Singapore Ministry of Health (MOH) sent out circulars to hospitals, government primary care clinics and medical practitioners to apprise them of the dengue situation during epidemic periods, and to provide periodic updates on guidelines for the management of dengue [14–19]. In 2007, a medical expert committee established the criteria for immediate referral to hospitals, which comprised objective criteria such as significant bleeding, fall in blood pressure, dehydration and/or postural hypotension, a rise in the haematocrit ≥ 20% above the baseline and platelet count < 80 000 cells / mm^3^, as well as subjective criteria such as severe vomiting or diarrhoea, severe abdominal pain, and elderly patients with medical co-morbidities who are unwell [15]. Another comprehensive set of hospital referral and admission criteria was also instituted during the 2013 dengue epidemic, and a key change to outpatient management of dengue was the platelet threshold of <60,000 / mm^3^ in adults and <80,000 / mm^3^ in children [17]. Besides the warning signs and symptoms listed in WHO’s dengue guidelines for diagnosis, treatment, prevention and control in 2009 [20] when considering referral to the hospital, MOH recommended to consider additional factors such as persistent fever, dizziness and platelet thresholds [17]. In 2015, the platelet threshold was lowered to <50,000 / mm^3^ in adults with no warning signs [18].

In this study, we describe the national trend of hospital admissions for dengue and disease severity in terms of length of stay (LOS), admission to the intensive care unit (ICU), death in hospital, and case fatality rate (CFR) over a 15-year period from 2003 to 2017. This will provide a baseline for future in-depth analyses of risk factors associated with adverse outcome to inform local guidelines for referral and hospitalization of dengue cases.

## Materials and methods

### Data sources

MOH provides clinical criteria for the diagnosis of dengue, and recommends appropriate laboratory tests and clinical management [21]. Under the Infectious Diseases Act in Singapore, it is mandatory for all medical practitioners and clinical laboratories to notify all clinically- or laboratory-confirmed dengue cases to MOH within 24 hours from the time of diagnosis through fax or via a dedicated website [21]. The information required in the notification form includes unique personal identification number, name, date of birth, ethnic group, gender, residential and school or workplace addresses, and dates of diagnosis and onset of illness. If a dengue case is notified to MOH from multiple sources (e.g., from clinician and laboratory), duplicate records are removed after verification checks based on personal particulars captured in the notifications. Laboratory confirmation of dengue cases is based on non-structural protein 1 (NS1) antigen detection, viral RNA detection by polymerase chain reaction (PCR), or immunoglobulin M detection [22,23].

Source reduction remains the key strategy to suppress the vector population in Singapore’s integrated *Aedes* mosquito control programme, and it entails house-to-house checks, vector surveillance, community education, law enforcement and operational research. An enhanced approach for dengue control has been adopted after a series of reviews of the programme in the past ten years, with focus on inter-epidemic surveillance and control, risk-based prevention and intervention, and coordinated intersectoral cooperation [24]. The National Environment Agency (NEA) is responsible for vector surveillance and control. Dengue serotype is determined at the Environmental Health Institute (EHI) of NEA and the National Public Health Laboratory based on residual blood samples tested positive for DENV from the national virus surveillance programme [8].

We conducted a retrospective study of notified cases and laboratory confirmed dengue patients admitted to all public and private acute hospitals between 2003 and 2017, so as to investigate the impact from the review and refinement of admission criteria. Inpatient information from all hospitals in Singapore is captured in the MediClaims database hosted by MOH, which contains electronic medical records that include up to three discharge diagnoses per patient based on the 9th and 10th revisions of the International Classification of Diseases (ICD). MOH conducts annual check on the MediClaims database to ensure its completeness.

We obtained the annual number of hospital admissions for all discharge diagnoses of dengue fever (DF) and dengue haemorrhagic fever (DHF) based on ICD-9 061 and 065.4 during 2003–2011 and ICD-10 A90 and A91 during 2012–2017. Hospitalizations with discharge diagnosis of dengue are mostly laboratory-confirmed, in accordance with the recommendations by MOH for initial evaluation of a patient suspected to have dengue [21]. Analyses of hospitalization data included all diagnosis types (principal or secondary) for DF and DHF. It must be noted that the principal cause of death for patients who died in hospital may not be due to dengue. For computation of CFR, the number of deaths due to dengue was obtained based on data from the Singapore Registry of Births and Deaths. Foreigners who came to Singapore to seek medical treatment were excluded from data analyses of dengue cases, hospitalizations and deaths.

### Data analysis

Annual incidence rates and hospitalization rates of dengue cases were calculated based on the estimated mid-year total population obtained from the Singapore Department of Statistics and expressed as per 100,000 population in a given year.

The Chi-square test for trend was used to evaluate the difference in proportions over time. We used two-sample independent z-tests to compare proportions between two groups for categorical variables. The Mann–Whitney U test was used to assess differences between any two groups for continuous variables. All statistical tests were two-sided, and statistical significance was taken as *p* < 0.05. Statistical analyses were performed using R version 3.5.1 (R Foundation for Statistical Computing, Vienna, Austria).

### Ethical statement

As the data used had been collected for the purpose of mandated national public health surveillance, ethics approval was not sought for the study. All data analyzed were anonymized.

## Results

During the 15-year study period, the dengue incidence rate per 100,000 population ranged from 47.9 in 2017 to 413.6 in 2013 (Table 1). The CFR ranged from 0% in 2017 to 0.27% in 2006. The proportion of dengue cases hospitalized plummeted from 97.3% in 2003 to an all-time low of 25.6% in 2014 (*p* < 0.0005). The proportion of deaths among hospitalized patients ranged from 0.14% in 2003 to 0.77% in 2009. The median age of fatal dengue cases ranged from 31 to 74 years, while the median age of dengue patients who died in hospital ranged from 52 to 86 years.

**Table 1:** Predominant circulating DENV serotype, dengue incidence rate, number of dengue deaths, case-fatality rate (%), proportion of dengue cases hospitalized and proportion of ICU admissions and deaths among hospitalized patients, 2003–2017*.

Year	Predominant circulating DENV serotype	Number of dengue cases per 100,000 population	Number of deaths due to dengue	Case fatality rate (%)	% of dengue cases hospitalized	% admitted to the ICU among hospitalized patients	% of deaths among hospitalized patients
2003	DENV-2	111.2	6	0.13	97.3	0.7	0.14
2004	DENV-1	230.1	8	0.08	88.8	0.6	0.15
2005	DENV-1	323.6	26	0.19	96.3	0.7	0.17
2006	DENV-1	66.8	8	0.27	72.6	1.0	0.61
2007	DENV-2	187.2	20	0.23	58.1	1.1	0.62
2008	DENV-2	140.4	10	0.15	47.0	1.3	0.41
2009	DENV-2	87.2	8	0.18	50.6	1.5	0.77
2010	DENV-2	103.1	4	0.08	47.9	0.8	0.32
2011	DENV-2	100.9	5	0.10	43.0	0.9	0.31
2012	DENV-2	86.7	2	0.04	41.9	1.1	0.41
2013	DENV-1	413.6	7	0.03	31.6	0.6	0.26
2014	DENV-1	327.0	5	0.03	25.6	0.9	0.20
2015	DENV-1 & 2	202.4	4	0.04	29.9	1.0	0.18
2016	DENV-2	231.5	9	0.07	28.6	0.6	0.35
2017	DENV-2	47.9	0	0.00	35.9	1.3	0.31

* Exclude foreigners who came to Singapore to seek medical treatment.

**Fig 1:**
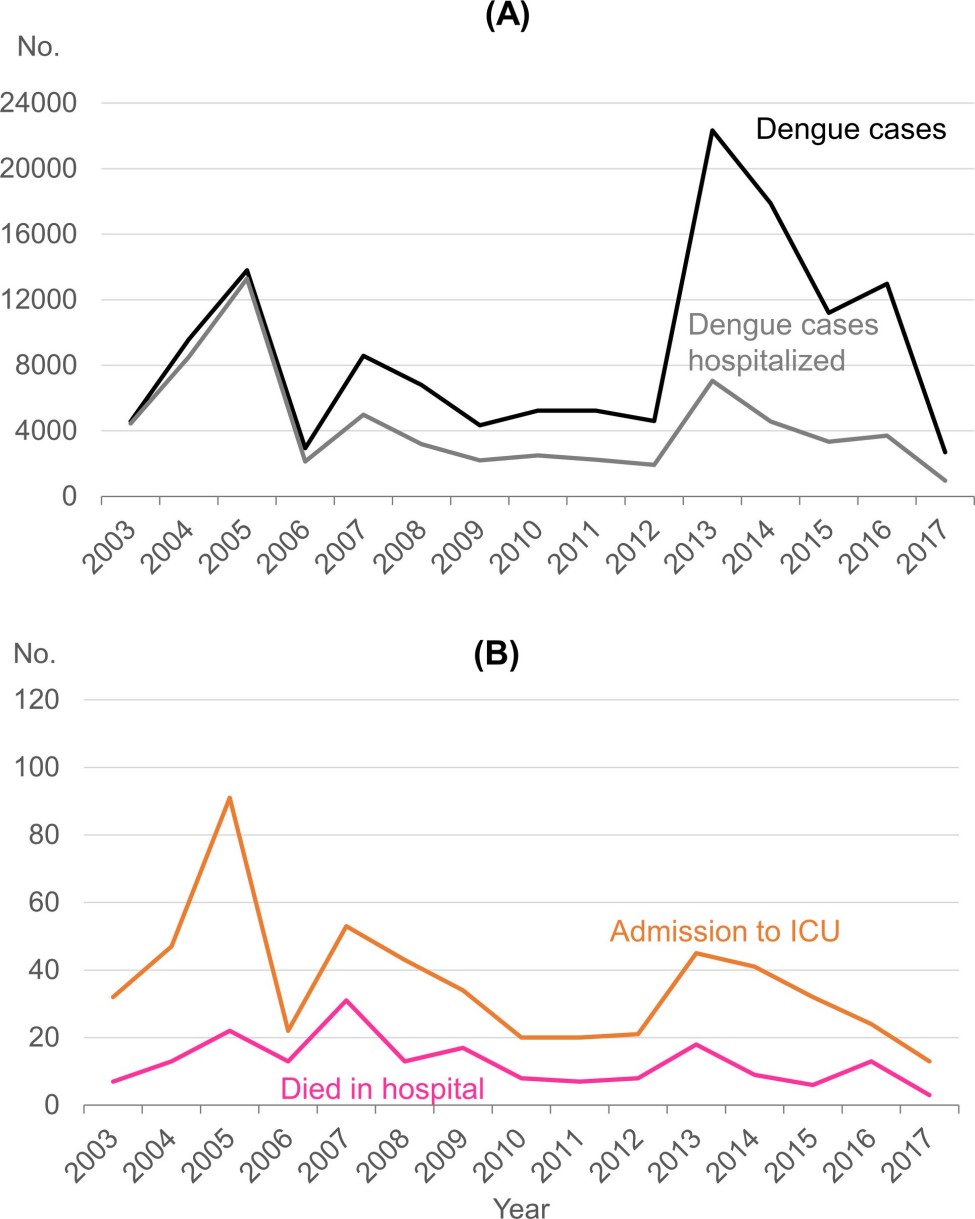
Number of (A) reported dengue cases and hospitalizations and (B) admissions to the ICU and deaths in hospital, 2003–2017.* Exclude foreigners who came to Singapore to seek medical treatment.

There were two large epidemics, each stretching over two years, in 2004–2005 and 2013–2014, both of which were associated with a switch in the predominant serotype from DENV-2 to DENV-1 (Table 1). Another epidemic in 2007 was associated with a switch from DENV-1 to DENV-2. The proportion of dengue cases hospitalized during these three epidemic periods was 93.2% in 2004–2005, 58.1% in 2007 and 28.9% in 2013–2014. While the 2004–2005 epidemic saw the largest proportion of cases hospitalized, a significant decline in the proportion hospitalized was observed in ensuing years (*p* < 0.0005) (Fig 1 and Table 1).

The gender-specific proportion of hospitalizations among dengue cases was consistently higher for women than for men during the study period, with the exceptions being in 2003 and 2006 (S1 Table). More dengue cases of older age were hospitalized (S1 Table); the highest proportions were in older adults aged 45–64 years from 2003 to 2012 (range: 50.8% to 100.0%) and elderly persons aged ≥65 years from 2013 to 2017 (range: 47.2% to 55.4%). In the majority of years after 2007, the hospitalization rate per 100,000 population was highest in the elderly ≥65 years of age.

**Fig 2:**
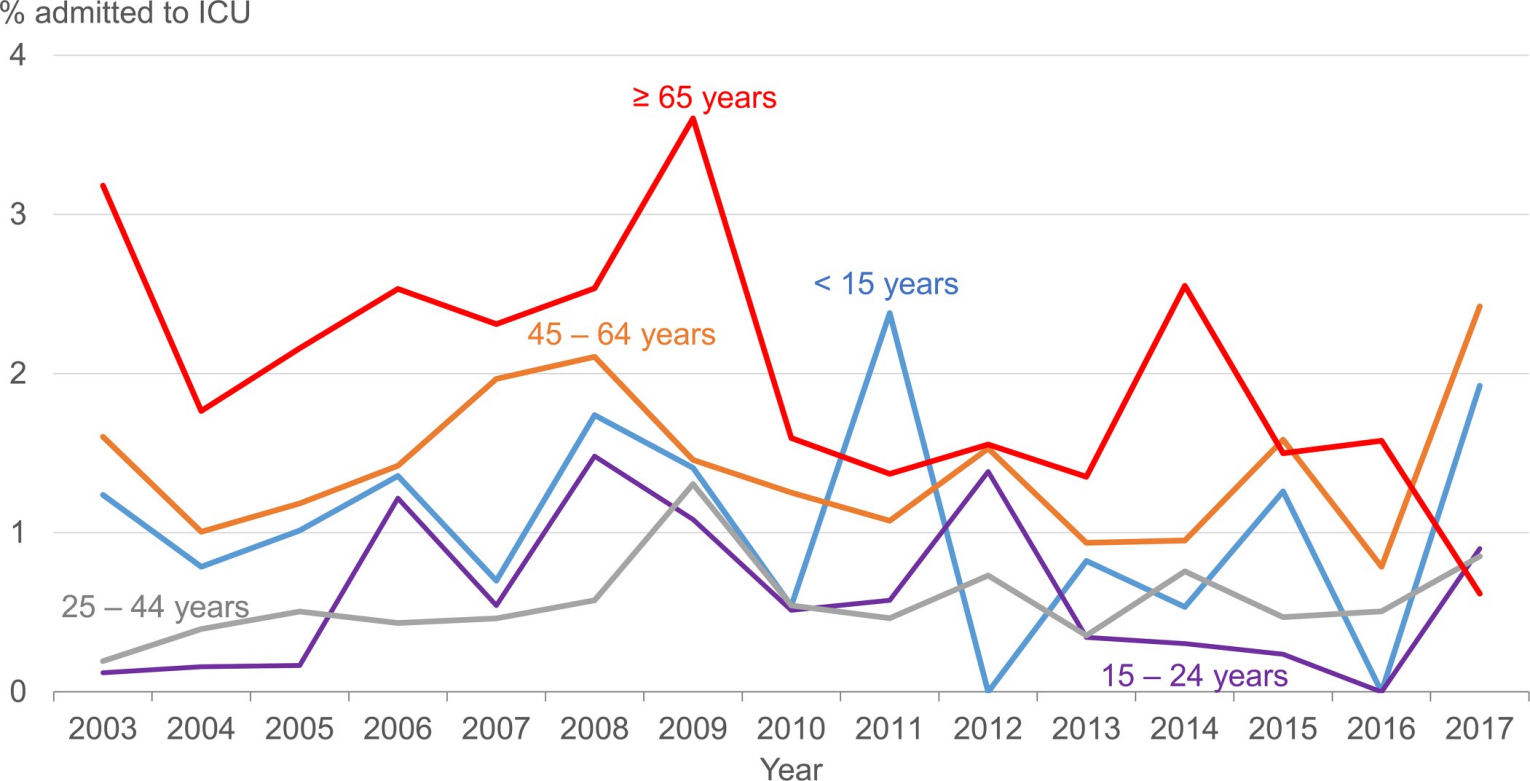
Proportion of dengue patients admitted to the ICU in hospital by age group, 2003–2017. * Exclude foreigners who came to Singapore to seek medical treatment.

About 0.6% to 1.5% of dengue patients in hospital were admitted to the ICU during the study period (Table 1). In the majority of years, this proportion was highest in elderly patients ≥65 years of age (range 0.6% to 3.6%) followed by older adults aged 45–64 years (range 0.8% to 2.4%) (Fig 2).

**Fig 3:**
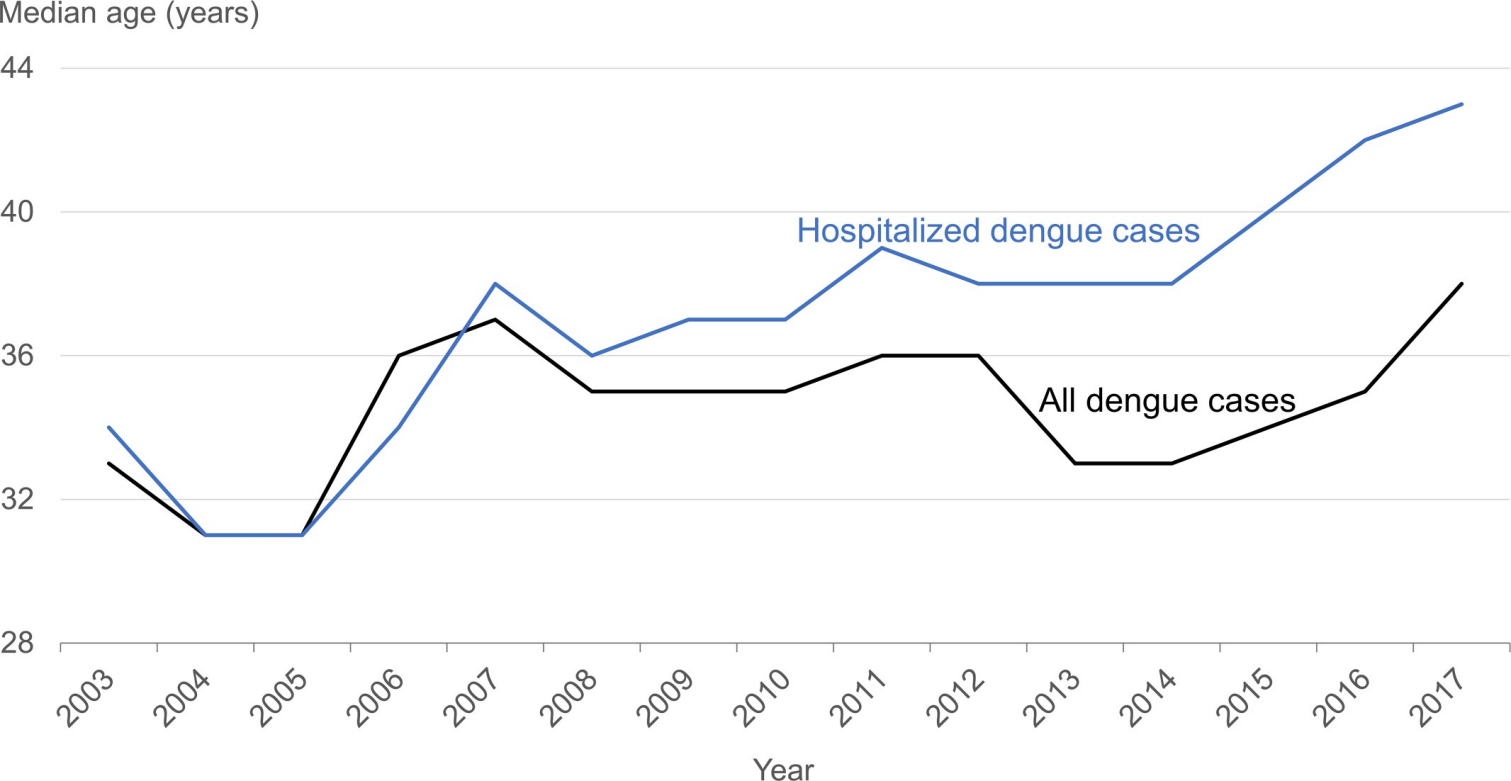
Median age of all dengue cases and hospitalized cases, 2003–2017. * Exclude foreigners who came to Singapore to seek medical treatment.

Men constituted 58.2% to 65.4% of dengue cases (S2 Table) and 53.9% to 61.2% of hospitalizations (S3 Table). Adults aged 25–44 years comprised about 40% to 50% of dengue cases and hospitalizations. While the proportion of hospitalizations in those aged 25–44 years declined from 46.3% in 2003 to 36.4% in 2017, older adults (aged ≥45 years) accounted for an increasingly higher proportion over the 15-year period: from 21.0% to 29.9% in the age group 45–64 years, and from 4.9% to 16.8% in elderly patients ≥65 years of age (all *p* < 0.0005) (S3 Table). The median age of dengue cases remained about the same from 2007 onwards (Fig 3). On the other hand, the median age among hospitalized cases increased from 38 years in 2007 to 43 years in 2017 (*p* < 0.0005) (Fig 3).

**Fig 4:**
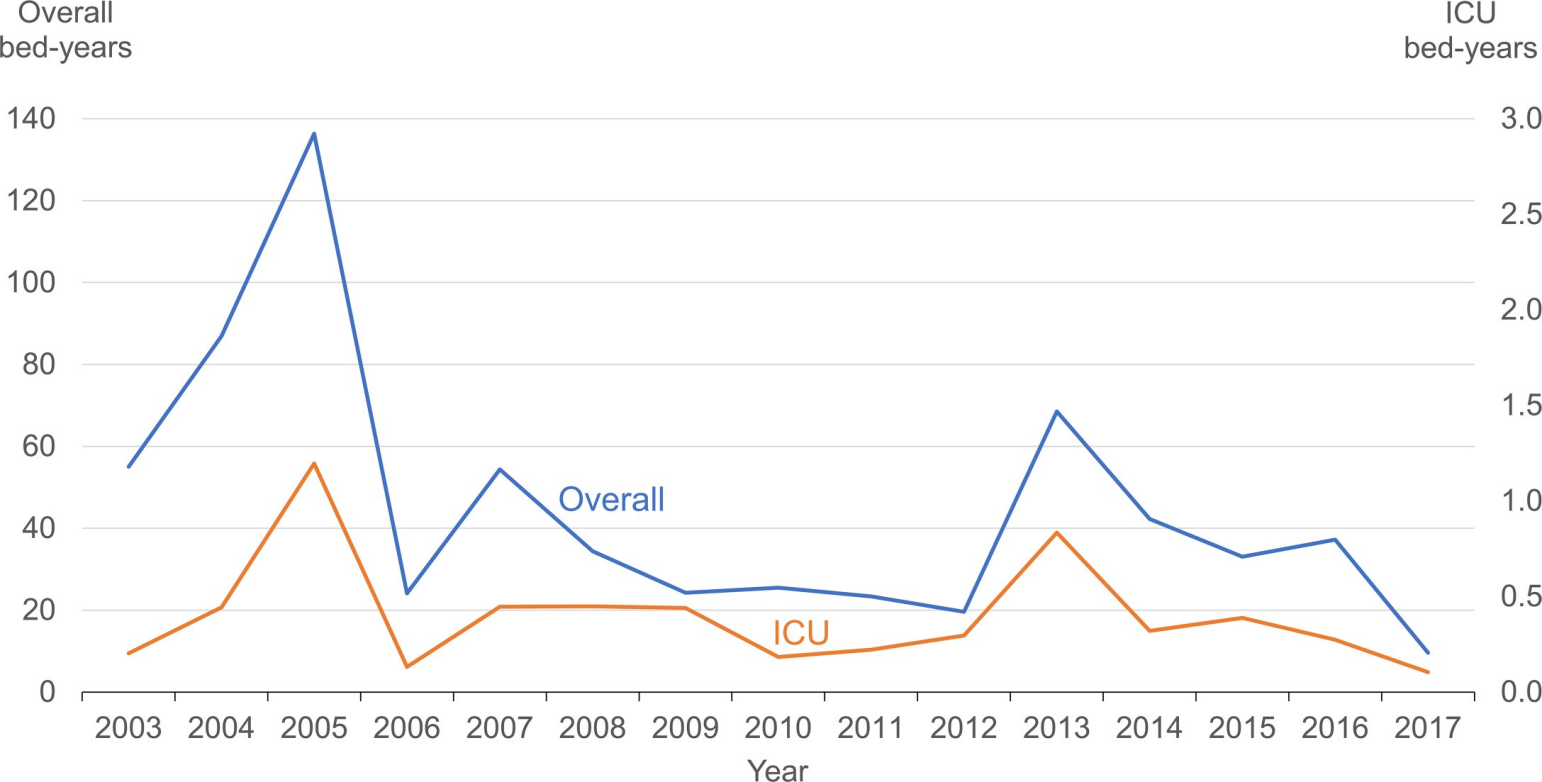
Number of bed-years of overall hospitalization and ICU stay for dengue patients, 2003–2017. * Exclude foreigners who came to Singapore to seek medical treatment.

The total number of hospital bed-years due to dengue halved from 136 in 2005 to 69 in 2013 (Fig 4). During the period from 2003 to 2017, the overall mean LOS in hospital was 3.8 days (range 1 to 121 days) and the overall median was 3 days (interquartile range [IQR] 2 to 5 days). The LOS in hospital remained stable over the years; both the annual mean and median LOS were 3 to 4 days. During the 15-year period, the overall mean ICU stay was 4.0 days (range 0 to 65 days) and the overall median was 2 days (IQR 1 to 4 days). The annual mean ICU stay was 2 to 7 days and the annual median was 2 to 3 days. Both the annual mean and annual median LOS in hospital for the age group 25–44 years was 3 to 4 days. The annual mean LOS for older adults aged 45–64 years was 3 to 5 days and the annual median was 3 to 4 days. Hospital stays among elderly patients ≥65 years of age were significantly longer: the annual mean LOS was 5 to 8 days and the annual median was 4 to 6 days (*p* < 0.0005).

## Discussion

The proportion of dengue cases hospitalized saw a sharp reduction in recent years despite the larger scale of dengue epidemics. The proportion hospitalized in the last five years of the study period ranged from 25.6% (in 2014) to 35.9% (in 2017), which was significantly lower than the 41.9% (in 2012) to 50.6% (in 2009) in the five-year period after the 2007 epidemic (Table 1). While there was a drastic decline in the proportion of dengue cases hospitalized due to refinement of hospital admission criteria in the aftermath of the 2004–2005 epidemic, there has been no concomitant increase in adverse outcomes, indicating that patients with more severe dengue requiring inpatient care and monitoring are being accurately identified and appropriately referred and admitted. Over the 15-year period, the proportion of patients admitted to the ICU remained below 2%, and the proportion who died in hospital was less than 1%.

Early diagnosis of dengue was facilitated by NEA’s EHI, which offered NS1 antigen testing to primary care clinics at no cost from 2006 [25]. A series of annual educational seminars targeted at primary care practitioners has been held since 2011 in conjunction with the ASEAN Dengue Day. These initiatives facilitate early diagnosis and close monitoring of dengue in the primary healthcare setting [23,26]. Surveys of primary care practices over two time periods in 2011 and 2014 demonstrated increased confidence and better management of dengue among primary care practitioners, with fewer referrals to hospital [26,27].

Despite the implementation of more selective admission criteria by the hospitals, we did not observe a concomitant increase in adverse outcomes nationally, as shown by the overall CFR (Table 1). This may be attributed to a combination of factors, including early diagnosis, improved dengue clinical management in primary care settings, and more appropriate referral and hospital admission [11,25–27]. Early diagnosis and right-siting of dengue case management can help to optimize usage of limited healthcare resources while averting negative outcomes. The new admission criteria implemented in a tertiary care public hospital in 2007 resulted in a median cost saving of US$1.4 million (90th percentile US$2.7 million) to patients in 2008 [13].

The impact of dengue epidemics on bed utilization rates was considerable even with the significant reduction in the proportion of dengue cases hospitalized. In the non-epidemic year 2012, there were 1,931 hospitalizations accounting for 20 bed-years (Fig 4). In comparison, there were 7,054 and 4,572 hospitalizations accounting for 69 and 42 bed-years in the most recent epidemic years of 2013 and 2014 respectively (Fig 4).

Our study revealed that a higher proportion of older dengue cases (aged ≥45 years) were admitted, and that older patients experienced more severe disease. The gap in median age between all dengue cases and hospitalized cases has widened since 2009 (Fig 3). Elderly patients ≥65 years of age comprised 17% of dengue hospitalizations in 2017, compared to 5% in 2003 (S3 Table). This was likely contributed to by both the MOH guideline to refer for hospital evaluation those aged ≥65 years as well as a shift in demographic profile of dengue cases towards older adults [17–19]. The proportion of dengue cases hospitalized among the elderly aged ≥65 years has been highest since 2013 (ranging from 47% to 55%) (S1 Table). The proportion of dengue patients who died in hospital was also highest in the elderly, while the proportion admitted to the ICU in elderly patients was one of the highest of any age group. The ageing population has led to additional challenges in the clinical management of dengue cases [28]. A retrospective study of all adult dengue patients managed at a tertiary care public hospital between 2005 and 2008 found that elderly patients had atypical clinical presentations and were at higher risk of DHF, severe disease and hospital acquired infection (HAI) [29]. The factors contributing to prolonged LOS were dengue severity, age, comorbidity and HAI [29].

Our study revealed that the implementation of more selective admission criteria by the hospitals had not led to longer LOS; the overall mean was 3.8 days and the overall median was 3 days (IQR 3 days) from 2003 to 2017. The overall proportion of hospitalized dengue cases who died over the 15-year period was 0.29%. In Malaysia, notifications for dengue infection represent only a small fraction of dengue incident cases (0.7% to 2.3%), and the proportion of dengue hospitalizations among estimated incident cases was about 3.0% to 5.6% based on data from 2001 to 2013 [30]. The mean LOS of dengue cases admitted to a tertiary care teaching hospital in Kelantan state of Malaysia during a six-year period from 2008 to 2013 was 4.88±2.74 days (median 3, IQR 3, range 1–35 days), and 1.1% of the hospitalized cases died [31]. A retrospective cohort study using the National Inpatient Sample, the largest all-payer database of hospital discharges in the United States from 2000 to 2007, found that the median LOS of hospitalized patients diagnosed with dengue was 3 days, with a range from 0 to 35 days [32].

A local study found that there has been a shift in the health-seeking behaviour of patients with dengue towards primary care: the proportion of dengue cases who sought medical attention at primary care clinics increased significantly from 14.8% in 2006 to 35.2% in 2015, while those who sought hospital care declined from 71.7% to 48.8% [25]. We believe this contributed to the significantly lower proportion of dengue cases admitted to hospital after the 2007 epidemic as more dengue cases were appropriately treated and monitored by primary care physicians. Another study reported an increase in the proportion of DHF from 6% in 2004 to 21% in 2007 among adult dengue patients admitted to a tertiary care public hospital, which suggested an improved triage system that accurately identified those patients requiring inpatient care and monitoring [33].

The main strength of this study lies in the use of data from a national surveillance system for notification of dengue cases and a hospitalization database hosted by MOH for the purpose of capturing all patient discharge information submitted by accredited institutions (both public and private).

There are a few limitations to our study. Clinical data of hospitalized dengue cases were not available for analysis in our study. Using WHO case classification criteria for diagnosis of DHF published in 1997 [34], a considerable proportion of DHF was either notified or diagnosed as DF, thereby its value as an indicator of disease severity was reduced. A systematic review involving studies in different countries and expert consensus meetings has suggested that the 1997 classification of DF, DHF and dengue shock syndrome (DSS) does not fully represent levels of disease severity [35]. We therefore opted to measure disease severity only in terms of admission to the ICU and deaths. While we could not determine if there had been delay in admission of dengue cases due to the more selective admission criteria resulting in adverse outcomes, those with more severe condition would most likely end up in hospital since there is good access to different levels of healthcare services in Singapore. Moreover, all dengue deaths reported to MOH are investigated and cross-checked with death data from the Singapore Registry of Births and Deaths, regardless of whether these fatal cases have been hospitalized or not. Comparison of trends may be limited by changes in laboratory tests for dengue and diagnostic practices over time and the advent of rapid laboratory diagnostic tools in later years. While the commonly used NS1 antigen assay was found to have high specificity, its sensitivity was significantly lower particularly in secondary infection [36]. During the dengue epidemic in 2007, cross-sectional seroepidemiologic surveys conducted in seven outbreak areas in Singapore revealed an overall inapparent dengue rate of 78% [37]. This study using case surveillance notifications does not capture all DENV infections. The proportion of persons with dengue who were hospitalized would be lower if inapparent dengue infections were included instead of only symptomatic dengue cases notified to MOH. There may be hospital discharges for dengue which were not notified to MOH, but this proportion is expected to be small as dengue is a notifiable disease under the Infectious Diseases Act.

In conclusion, the drastic decline in proportion of dengue cases hospitalized due to more selective admission criteria has not led to a corresponding increase in adverse consequences. Our study suggests that following the nadir in the proportion of dengue cases hospitalized after 2007 of about 26%, the subsequent increase after the 2013–2014 dengue epidemic was likely contributed to by both an increased risk of disease among the older population as well as the inclusion of age ≥65 years as a specific indication for hospitalization. In the light of Singapore’s ageing population and high hospital bed occupancy rates, coupled with periodic dengue epidemics, it is important to manage unexpected surges in dengue cases and ameliorate the strain on hospital systems with appropriate referral for treatment and right-siting of care. Further studies are needed to improve dengue management in older adults and further identify risk factors of severe disease in this age group.

## Supporting information

S1 Checklist STROBE checklist. (DOC)

S1 Table Gender-specific and age-specific proportion (%) of dengue cases hospitalized, 2003–2017. (DOCX)

S2 Table Distribution (%) of dengue cases by gender, age group, residency and ethnic group, 2003–2017. (DOCX)

S3 Table Distribution (%) of dengue hospitalizations by gender, age group, residency and ethnic group, 2003–2017. (DOCX)
