# Supplementary material for: A 15-year review of dengue hospitalizations in Singapore: Reducing admissions without adverse consequences, 2003 to 2017
Source: PLoS Negl Trop Dis. 2019 May 15;13(5):e0007389. doi: 10.1371/journal.pntd.0007389 (PMC6519799; doi:10.1371/journal.pntd.0007389)
Supplement: S1 Table — (DOCX) [file pntd.0007389.s002.docx]

**S1 Table. Gender-specific and age-specific proportion (%) of dengue cases hospitalized, 2003-2017.***

|  | **Year** | | | | | | | | | | | | | | | | | | | | | | | | | | |
| --- | --- | --- | --- | --- | --- | --- | --- | --- | --- | --- | --- | --- | --- | --- | --- | --- | --- | --- | --- | --- | --- | --- | --- | --- | --- | --- | --- |
|  | **2003** | **2004^╪^** | | **2005^╪^** | | **2006** | | **2007^╪^** | | **2008** | | **2009** | | **2010** | **2011** | | **2012** | | **2013^╪^** | | **2014^╪^** | | **2015** | | **2016** | | **2017** |
| **Gender** |  |  | |  | |  | |  | |  | |  | |  |  | |  | |  | |  | |  | |  | |  |
| Male | 98.1 | 82.8 | | 95.3 | | 74.7 | | 57.1 | | 46.4 | | 48.9 | | 45.4 | 40.7 | | 39.1 | | 28.5 | | 22.4 | | 27.2 | | 25.8 | | 33.6 |
| Female | 96.3 | 98.4 | | 97.7 | | 69.6 | | 59.5 | | 48.0 | | 53.3 | | 51.8 | 46.3 | | 46.7 | | 37.2 | | 31.5 | | 34.4 | | 32.7 | | 39.4 |
|  |  |  | |  | |  | |  | |  | |  | |  |  | |  | |  | |  | |  | |  | |  |
| **Age group** | | |  | |  | |  | |  | |  | |  | | |  | |  | |  | |  | |  | |  | |
| 0–14 | 87.6 | 77.7 | | 83.4 | | 81.0 | | 44.0 | | 46.7 | | 50.7 | | 46.4 | 41.8 | | 41.0 | | 33.8 | | 27.5 | | 31.9 | | 25.6 | | 30.6 |
| 15–24 | 97.4 | 89.2 | | 100.0 | | 86.9 | | 55.9 | | 45.9 | | 46.5 | | 44.6 | 40.3 | | 37.7 | | 27.9 | | 20.8 | | 22.9 | | 21.8 | | 32.6 |
| 25–34 | 96.2 | 84.5 | | 96.1 | | 77.2 | | 56.5 | | 42.7 | | 44.9 | | 43.2 | 36.3 | | 34.5 | | 24.1 | | 19.9 | | 22.7 | | 20.4 | | 27.3 |
| 35–44 | 100.0 | 89.3 | | 97.3 | | 80.9 | | 59.3 | | 48.1 | | 53.8 | | 44.6 | 41.1 | | 43.7 | | 29.1 | | 22.4 | | 26.1 | | 25.9 | | 34.9 |
| 45–54 | 100.0 | 100.0 | | 100.0 | | 81.3 | | 67.7 | | 55.0 | | 57.1 | | 58.2 | 49.3 | | 47.7 | | 38.5 | | 29.6 | | 36.4 | | 32.5 | | 38.5 |
| 55–64 | 100.0 | 100.0 | | 100.0 | | 60.7 | | 69.7 | | 56.7 | | 58.7 | | 59.6 | 56.7 | | 55.1 | | 46.5 | | 43.8 | | 42.7 | | 45.4 | | 46.9 |
| 65+ | 100.0 | 99.4 | | 100.0 | | 35.3 | | 50.4 | | 40.0 | | 49.9 | | 52.8 | 45.4 | | 45.3 | | 49.4 | | 48.1 | | 55.4 | | 55.4 | | 47.2 |

* Exclude foreigners who came to Singapore to seek medical treatment.

**^╪^** Dengue epidemic years.
