# Supplementary material for: A 15-year review of dengue hospitalizations in Singapore: Reducing admissions without adverse consequences, 2003 to 2017
Source: PLoS Negl Trop Dis. 2019 May 15;13(5):e0007389. doi: 10.1371/journal.pntd.0007389 (PMC6519799; doi:10.1371/journal.pntd.0007389)
Supplement: S2 Table — (DOCX) [file pntd.0007389.s003.docx]

**S2 Table. Distribution (%) of dengue cases by gender and age group, 2003-2017.***

|  | **Year** | | | | | | | | | | | | | | | | | | | | | | | | | | |
| --- | --- | --- | --- | --- | --- | --- | --- | --- | --- | --- | --- | --- | --- | --- | --- | --- | --- | --- | --- | --- | --- | --- | --- | --- | --- | --- | --- |
|  | **2003** | **2004^╪^** | | **2005^╪^** | | **2006** | | **2007^╪^** | | **2008** | | **2009** | | **2010** | **2011** | | **2012** | | **2013^╪^** | | **2014^╪^** | | **2015** | | **2016** | | **2017** |
| **Gender** |  |  | |  | |  | |  | |  | |  | |  |  | |  | |  | |  | |  | |  | |  |
| Male | 58.2 | 61.6 | | 57.7 | | 59.5 | | 59.6 | | 61.1 | | 60.7 | | 60.8 | 59.3 | | 62.6 | | 64.0 | | 65.4 | | 62.6 | | 59.6 | | 58.6 |
| Female | 41.8 | 38.4 | | 42.3 | | 40.5 | | 40.4 | | 38.9 | | 39.3 | | 39.2 | 40.7 | | 37.4 | | 36.0 | | 34.6 | | 37.4 | | 40.4 | | 41.4 |
|  |  |  | |  | |  | |  | |  | |  | |  |  | |  | |  | |  | |  | |  | |  |
| **Age group** | | |  | |  | |  | |  | |  | |  | | |  | |  | |  | |  | |  | |  | |
| 0–14 | 10.1 | 12.0 | | 13.7 | | 9.3 | | 7.6 | | 7.3 | | 6.4 | | 7.6 | 7.7 | | 5.7 | | 6.4 | | 7.6 | | 6.7 | | 6.8 | | 6.3 |
| 15–24 | 18.7 | 22.2 | | 21.8 | | 16.1 | | 15.3 | | 17.3 | | 18.2 | | 16.7 | 16.5 | | 16.6 | | 18.8 | | 17.8 | | 16.5 | | 16.0 | | 12.6 |
| 25–34 | 24.6 | 25.8 | | 23.2 | | 21.3 | | 22.0 | | 24.1 | | 23.6 | | 24.1 | 22.7 | | 25.2 | | 27.1 | | 27.8 | | 26.9 | | 26.1 | | 24.7 |
| 35–44 | 21.4 | 19.9 | | 19.8 | | 18.5 | | 21.6 | | 21.1 | | 19.6 | | 23.9 | 20.1 | | 20.9 | | 20.9 | | 21.4 | | 20.3 | | 20.5 | | 18.2 |
| 45–54 | 13.9 | 11.0 | | 11.3 | | 12.0 | | 13.6 | | 12.2 | | 12.7 | | 13.2 | 14.5 | | 13.0 | | 13.2 | | 13.4 | | 13.9 | | 14.4 | | 13.8 |
| 55–64 | 6.6 | 5.5 | | 5.8 | | 7.6 | | 8.9 | | 7.9 | | 9.1 | | 7.6 | 9.4 | | 9.3 | | 7.6 | | 7.1 | | 9.3 | | 9.1 | | 11.6 |
| 65+ | 4.8 | 3.6 | | 4.4 | | 15.2 | | 11.0 | | 10.2 | | 10.2 | | 6.8 | 9.2 | | 9.2 | | 6.0 | | 5.0 | | 6.4 | | 7.0 | | 12.8 |

* Exclude foreigners who came to Singapore to seek medical treatment.

**^╪^** Dengue epidemic years.
