# Supplementary material for: A 15-year review of dengue hospitalizations in Singapore: Reducing admissions without adverse consequences, 2003 to 2017
Source: PLoS Negl Trop Dis. 2019 May 15;13(5):e0007389. doi: 10.1371/journal.pntd.0007389 (PMC6519799; doi:10.1371/journal.pntd.0007389)
Supplement: S3 Table — (DOCX) [file pntd.0007389.s004.docx]

**S3 Table. Distribution (%) of dengue hospitalizations by gender and age group, 2003-2017.***

|  | **Year** | | | | | | | | | | | | | | | | | | | | | | | | | | | |
| --- | --- | --- | --- | --- | --- | --- | --- | --- | --- | --- | --- | --- | --- | --- | --- | --- | --- | --- | --- | --- | --- | --- | --- | --- | --- | --- | --- | --- |
|  | **2003** | | **2004^╪^** | | **2005^╪^** | | **2006** | | **2007^╪^** | | **2008** | | **2009** | | **2010** | **2011** | | **2012** | | **2013^╪^** | | **2014^╪^** | | **2015** | | **2016** | | **2017** |
| **Gender** |  | |  | |  | |  | |  | |  | |  | |  |  | |  | |  | |  | |  | |  | |  |
| Male | 58.7 | | 57.4 | | 57.0 | | 61.2 | | 58.5 | | 60.3 | | 58.6 | | 57.6 | 56.2 | | 58.3 | | 57.7 | | 57.4 | | 56.9 | | 53.9 | | 54.7 |
| Female | 41.3 | | 42.6 | | 43.0 | | 38.8 | | 41.5 | | 39.7 | | 41.4 | | 42.4 | 43.8 | | 41.7 | | 42.3 | | 42.6 | | 43.1 | | 46.1 | | 45.3 |
|  |  | |  | |  | |  | |  | |  | |  | |  |  | |  | |  | |  | |  | |  | |  |
| **Age group** | |  | |  | |  | |  | |  | |  | |  | | |  | |  | |  | |  | |  | |  | |
| 0–14 | 9.1 | | 10.5 | | 11.9 | | 10.3 | | 5.7 | | 7.2 | | 6.5 | | 7.4 | 7.5 | | 5.5 | | 6.9 | | 8.2 | | 7.1 | | 6.1 | | 5.4 |
| 15–24 | 18.7 | | 22.3 | | 22.7 | | 19.2 | | 14.8 | | 16.9 | | 16.8 | | 15.6 | 15.4 | | 15.0 | | 16.5 | | 14.4 | | 12.6 | | 12.1 | | 11.5 |
| 25–34 | 24.3 | | 24.6 | | 23.1 | | 22.6 | | 21.4 | | 21.9 | | 20.9 | | 21.8 | 19.2 | | 20.7 | | 20.7 | | 21.6 | | 20.4 | | 18.7 | | 18.7 |
| 35–44 | 22.0 | | 20.0 | | 20.0 | | 20.6 | | 22.1 | | 21.6 | | 20.8 | | 22.3 | 19.2 | | 21.7 | | 19.2 | | 18.7 | | 17.7 | | 18.6 | | 17.7 |
| 45–54 | 14.3 | | 12.4 | | 11.7 | | 13.4 | | 15.9 | | 14.3 | | 14.4 | | 16.1 | 16.6 | | 14.8 | | 16.0 | | 15.5 | | 16.9 | | 16.4 | | 14.8 |
| 55–64 | 6.7 | | 6.2 | | 6.1 | | 6.4 | | 10.6 | | 9.5 | | 10.6 | | 9.4 | 12.4 | | 12.3 | | 11.2 | | 12.1 | | 13.3 | | 14.4 | | 15.1 |
| 65+ | 4.9 | | 4.0 | | 4.5 | | 7.4 | | 9.5 | | 8.6 | | 10.1 | | 7.5 | 9.7 | | 10.0 | | 9.4 | | 9.4 | | 11.9 | | 13.7 | | 16.8 |

* Exclude foreigners who came to Singapore to seek medical treatment.

* Dengue epidemic years.
